# Identification of diagnostic biomarkers for relapsing-remitting multiple sclerosis in plasma by mass spectrometry-based proteomics

**DOI:** 10.1093/jnen/nlaf145

**Published:** 2025-12-22

**Authors:** Víctor M García-Silva, Eduardo Chicano-Galvez, María Isabel García-Sánchez, Ángela Peralbo-Molina, María Hernández-Valladares

**Affiliations:** Department of Physical Chemistry, University of Granada, Granada, Spain; Mass Spectrometry and Molecular Imaging Unit (IMSMI), Maimonides Biomedical Research Institute of Cordoba (IMIBIC), Reina Sofia University Hospital, University of Cordoba (UCO), Cordoba, Spain; Andalusian Public Health System (SSPA) Biobank, Sevilla node, Virgen Macarena University Hospital, Sevilla, Spain; Mass Spectrometry and Molecular Imaging Unit (IMSMI), Maimonides Biomedical Research Institute of Cordoba (IMIBIC), Reina Sofia University Hospital, University of Cordoba (UCO), Cordoba, Spain; Department of Physical Chemistry, University of Granada, Granada, Spain; Biosanitary Research Institute of Granada ibs.GRANADA, Granada, Spain

**Keywords:** biomarkers, extracellular vesicles, mass spectrometry, plasma, proteomics, relapsing remitting multiple sclerosis, von Willebrand factor

## Abstract

In the absence of molecular biomarkers, the current diagnosis of multiple sclerosis is based on clinical assessment, neuroimaging, and detection of oligoclonal bands in cerebrospinal fluid. Early and rapid diagnosis using patient samples obtained by non-invasive methods would be a major advance in clinical management. We tested 5 different methods for preparation of the plasma proteome for liquid chromatography with tandem mass spectrometry (LC-MS/MS) analysis. These were as follows: (1) single-pot, solid-phase-enhanced sample preparation (SP3), (2) iST and (3) ENRICH-iST of raw plasma, (4) SP3 of highly abundant-proteins depleted plasma (DEPL-SP3), and (5) SP3 of plasma extracellular vesicles (EV-SP3). DEPL-SP3 and EV-SP3 sample preparation workflows yielded the highest numbers of quantified plasma proteins. Using both methods, we analyzed the plasma proteome of 15 relapsing-remitting multiple sclerosis (RRMS) patients and 5 healthy controls. We found 54 and 35 regulated plasma proteins with DEPL-SP3 and EV-SP3 workflows, respectively. Among them, von Villebrand factor (VWF) was identified as a potential RRMS diagnostic biomarker. The use of sample preparation workflows for LC-MS/MS analysis that describe both the soluble and EV plasma proteomes might increase the likelihood of identifying new and robust RRMS biomarkers.

## INTRODUCTION

Multiple sclerosis (MS) is a chronic, autoimmune, neuroinflammatory, and neurodegenerative disorder of the central nervous system (CNS), which damages the myelin and lead to axonal and neuronal loss.^1^ MS is considered to be a multifactorial disease. There are associations with mutations in several genes such as *HLA DR15*, interleukin-7 (*IL7*), and interleukin-2 receptor alpha (*IL2RA*) and with environmental factors such as exposure to Epstein Barr virus, low levels of vitamin D, smoking, and diet.^2^^,^^3^ A recent study has shown that interferon gamma (IFNG)-mediated induction of the immunoproteasome subunit PSMB8 reduces proteasomal activity, resulting in accumulation of phosphofructo-2-kinase/fructose-2,6-bisphosphatase 3 (PFKFB3), enhanced neuronal glycolysis, oxidative injury, and ferroptosis in MS.^4^

The diagnosis of MS is based on the detection of objective evidence of dissemination in space and time of typical MS lesions, as well as the exclusion of another diagnostic parameters. In addition to the clinical assessment, radiological and laboratory tests including magnetic resonance imaging (MRI), analysis of cerebrospinal fluid (CSF), and visual evoked potentials (VEP) are used as diagnostic tools.^5^ The most recent revision of McDonald criteria for the diagnosis of MS suggested that the presence of CSF-specific oligoclonal bands (OCB) in the absence of atypical CSF findings allows for the diagnosis.^6^ Early diagnosis of MS is important as it allows treatment to be provided in the initial stages of the disease, thereby slowing down the progression and reducing the costs and burdens associated with the different disability levels of MS.^7^^,^^8^ Thus, the incorporation of advanced imaging and new body fluid biomarkers for the diagnosis and prognosis are key to MS treatment.

There are several technological approaches used to identify biomarkers in different biofluids such as CSF, plasma, or serum. Among them, liquid chromatography-tandem mass spectrometry (LC-MS/MS)-based proteomics is the most comprehensive and unbiased approach for the quantitative profiling of proteins. Despite new technological advances, the selection of best preparation workflows for complex proteomic matrixes such as plasma or serum for LC-MS/MS studies is challenging due to the presence of highly abundant proteins (eg, albumin). An increasing availability of new tools may enhance the ability to circumvent the effects of these components in proteomic analyses.

In the current study, several methods for plasma sample preparation were tested to determine which method yields the highest number of quantified proteins. These methods included commercial solutions for enrichment of low abundance proteins such as the high-select top14 abundant protein depletion resin and the novel ENRICH-iST kit as well as differential ultracentrifugation for the isolation of extracellular vesicles (EVs) followed by the single-pot, solid-phase-enhanced sample preparation (SP3) for protein capture and digestion of immunodepleted and EV samples.^9^ SP3 and iST methods with raw plasma were incorporated into the study as background procedures. Based on the results, the plasma proteomes of a group of 15 relapsing remitting multiple sclerosis (RRMS) patients at the time of diagnosis and 5 healthy controls (HCs) were then characterized by LC-MS/MS.

## METHODS

### Study population

Three out of four MS patients are women according to the Spanish Society of Neurology (SEN; www.sen.es). Because of this fact and fund limitations, this study focused on 15 women diagnosed with RRMS. Therefore, sex impact on protein profiling will not be addressed in the method testing approach in this study assessing RRMS proteome characterization methods. Most patients were diagnosed with RRMS between ages 23 and 48 years according to the diagnostic criteria established in the 2017 revision of the McDonald criteria^6^ (Table S1). Biological samples were collected at the time of diagnosis, prior to the administration of any treatment. Inclusion and exclusion criteria were established to ensure cohort homogeneity. Inclusion criteria included being of legal age, meeting the 2017 McDonald diagnostic criteria, and presenting positive oligoclonal bands in CSF. Exclusion criteria included being underage, having received treatments prior to sample collection, and the coexistence of diseases concomitant with MS. All the plasma samples were isolated at the University Hospital Virgen Macarena between 2021 and 2022. The control group consisted of 5 sex- and age-matched HCs (Table S2).

This study was approved by the ethics committee of the University Hospital Virgen Macarena and of the Coordinator of Biomedical Research in Andalusia. Signed informed consent was obtained from all the enrolled study subjects.

### Human plasma samples

Blood from 15 RRMS patients and 5 HCs, collected in tubes with K2-EDTA anticoagulant, was centrifugated after collection at 1500 × g for 10 minutes followed by a second centrifugation at 2500 × g for 15 minutes to achieve platelet-poor plasma. Plasma samples from the 5 HCs were also used for testing methods.

### Individual proteome sample preparation

Depletion of highly abundant plasma proteins from 10 µL of plasma was carried out with the high-select top 14 abundant protein depletion mini spin columns (ThermoFisher Scientific) according to the manufacturer’s instructions.

For the enrichment of EVs by ultracentrifugation, 500 µL of plasma samples were supplemented with phosphate-buffered saline (PBS) until the final volume reached 1000 µL. The samples were then centrifuged at 20 000 × g at 4°C for 30 minutes, and the supernatants were discarded, leaving 20 µL of buffer together with the EV pellets. EV pellets were supplemented with PBS to 1000 µL followed by a second centrifugation at 20 000 × g at 4°C for 30 minutes. Finally, the supernatants were removed and the pellets with a small volume (approximately 20 µL of PBS) were stored at −20°C.

Replicates of 20 µg of immunodepleted plasma, plasma EVs, and raw plasma were simultaneously reduced in 5 mM tris(2-carboxyethyl)phosphine (TCEP) and alkylated in 10 mM chloroacetamide (CAA) in the presence of 4% SDS at 95°C for 7 minutes before being digested with the SP3 method.^9^ Digestion was performed using a 1:1 ratio of magnetic carboxylated-modified beads (Cytiva).

One and 20 µL of raw plasma samples were also processed with the iST and ENRICH-iST technology (PreOmics), respectively. With the ENRICH-iST sample preparation kit, an additional protein enrichment step was performed before the standard iST sample processing protocol. Eluted peptides were lyophilized and stored at −20°C.

### Peptide cleaning

SP3-prepared peptides were acidified to pH 2-3 with trifluoroacetic acid (TFA). Desalting was performed using Oasis HLB 96-well µElution Plate, 30 mm (Waters), according to the manufacturer’s instructions. Peptide concentrations of all samples were determined using Nanodrop OneC and absorbance readings at 280 nm.

### LC-MS/MS analysis

Purified tryptic digests were randomized and separated with the predefined 60 SPD method (21 minutes gradient time, 200 ng peptides) on an Evosep One LC system (Evosep) coupled online to a tims-TOF Flex mass spectrometer (Bruker Daltonics) with dia-PASEF data acquisition mode. LC-MS/MS data raw files were analyzed with Spectronaut proprietary software (Spectronaut 18, Biognosys) with a directDIA approach, setting the entire sample dataset as the experimental set and using a fasta file containing the Uniprot human reference proteome to generate an in silico spectral library.

### Statistics and data processing

Data from each individual sample were normalized using the loess function from the limma package to correct for loading bias between samples and conditions.^10^ Proteins with at least four quantitative valid values in each of the comparing groups (RRMS and HC) were selected. Further normalization by median subtraction for each sample prior to Welch’s *t*-test was performed with the Perseus platform v2.0.22.^11^

Bar plots for the number of quantified proteins and peptides were created using GraphPad Prism 9.0.0 (GraphPad Software). The Bioinformatics and Evolutionary Genomics online tool (https://bioinformatics.psb.ugent.be/webtools/Venn/) and Venny tool (https://bioinfogp.cnb.csic.es/tools/venny/index.html) were utilized to create Venn diagrams. Gene ontology (GO) and Reactome pathways enrichment were done using Enrichr.^12^ Protein-protein interaction (PPI) networks were obtained by using the STRING database version 12.0 at a high confidence score of 0.7.^13^ Networks were visualized using the Cytoscape platform version 3.9.1.^14^

### Nanoparticle tracking analysis (NTA)

The size of plasma EVs enriched by 20 000 × g centrifugation was observed by NTA using a NanoSight nanoparticle analyzer (Malvern Panalytical) using the 488 nm laser. The instrument was calibrated using 100 nm polystyrene beads before sample analysis. Samples were diluted 1000 times in PBS pH 7.2 and measured at 27°C.

## RESULTS

**Figure 1.**
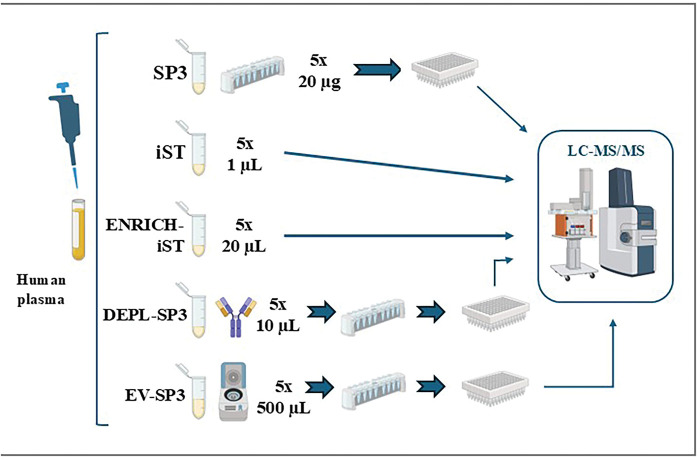
Experimental workflow for the testing of 5 methodologies, that is, single-pot, solid-phase-enhanced sample preparation (SP3), iST and ENRICH-iST of raw plasma; SP3 with highly abundant-proteins depleted plasma (DEPL-SP3); and SP3 with plasma extracellular vesicles (EV-SP3) for the analysis of plasma proteome by LC-MS/MS. iST and ENRICH-iST protocols were processed according to the corresponding PreOmics kits. The number of replicates (5×) and protein amount or volume of plasma are indicated for each approach. The picture was created with elements from BioRender.

In this study, we compared the performance of the SP3 protocol and commercial iST and ENRICH-iST kits in the preparation of raw human plasma proteomes. Furthermore, we have studied protein identification and quantification, using the SP3 workflow, in immunodepleted plasma (DEPL-SP3) and plasma enriched extracellular vesicles (EV-SP3). All the proteomes were analyzed using LC-MS/MS (Figure 1). Methods giving the deepest proteome coverage were used for plasma diagnostics of an RRMS cohort of 15 patients.

### Method testing

**Figure 2.**
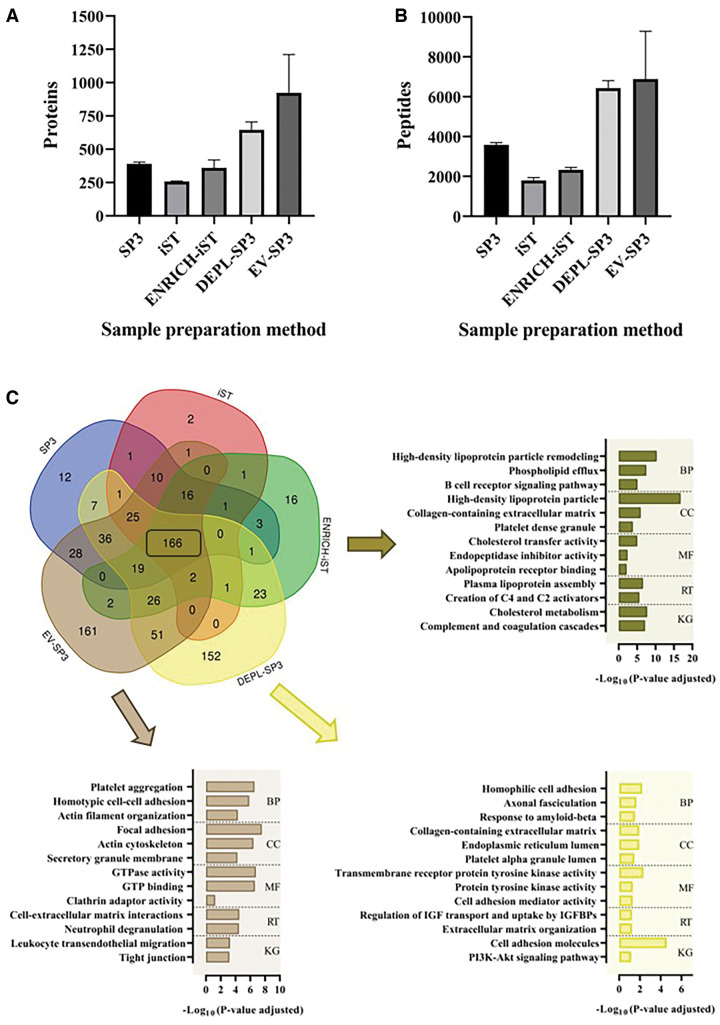
Human plasma quantified proteins (A) and peptides (B) using SP3, iST, and ENRICH-iST with raw plasma; SP3 with highly abundant-proteins depleted plasma, DEPL-SP3, and SP3 with plasma extracellular vesicles, EV-SP3. Venn diagram of the proteins quantified in the 5 sample processing workflows (C). Enrichment of the GO terms, Reactome terms and KEGG pathways of the 166 overlapping proteins using protein datasets from the 5 methodologies and the 152 and 151 proteins exclusively quantified by the DEPL-SP3 and EV-SP3 methodologies, respectively, are shown in bar plots.

Among the sample preparation strategies using raw plasma from 5 HCs, the SP3 protocol outperformed the iST and ENRICH-iST workflows, both in the identification and quantification of proteins and peptides (391 ± 6 and 3591 ± 46 as mean ± SEM, respectively) (Figure 2A and B). The combination of the immunodepletion step followed by the SP3 protocol (DEPL-SP3) provided with an even higher number of identified and quantified proteins and peptides (646 ± 27; 6428 ± 169). Nevertheless, the use of the EV enrichment step by ultracentrifugation followed by the SP3 protocol (EV-SP3) yielded the highest numbers of quantified proteins and peptides (923 ± 130; 6884 ± 1073). All the procedures produced a very similar number of quantified proteins in 4 HC plasma samples (Figure S1). However, the remaining HC sample showed an unexpectedly higher number of plasma proteins throughout the 5 methodologies.

To further characterize the human plasma proteomes identified and quantified by the different strategies, Venn diagrams were plotted using the list of common proteins observed in every replicate for each of the procedures (Figure 2C; Figure S1). One hundred sixty-six proteins were quantified by all 5 methodologies. GO term analyses showed that high-density lipoprotein particle remodeling and B cell receptor signaling pathway were significantly enriched biological processes in the samples quantified with all the 5 methodologies. These enriched proteins were primarily located in high-density lipoprotein particles. Reactome terms and KEGG pathway analyses showed a significant enrichment of proteins involved in plasma lipoprotein assembly, cholesterol metabolism, and complement cascades (Figure 2C). In addition to these proteins, 152 and 161 were only quantified by the DEPL-SP3 and the EV-SP3 methodology, respectively. GO term analysis of DEPL-SP3-quantified proteins showed association to homophilic cell adhesion, axonal fasciculation, and response to amyloid-beta biological processes and to tyrosine kinase activity molecular functions. KEGG pathway cell adhesion molecules were significantly enriched in this set of proteins. Regarding the proteins exclusively quantified by the EV-SP3 procedure, which allowed the isolation of large EVs with a median size of 192 nm (Figure S2) (ie, microvesicles^15^), a significant enrichment of biological processes such as platelet aggregation and cell-cell adhesion was observed (Figure 2C). Furthermore, EV-SP3 proteins were associated with GTPase activity, with focal adhesion and secretory granule membrane as cell compartment and with cell-extracellular matrix interactions and neutrophil degranulation as Reactome terms.

Taken together, enrichment of plasma proteins by either immunodepletion or EV enrichment protocols notably improved the number of protein identifications and quantifications. Both techniques jointly quantified the majority of the proteins observed by all the 5 testing workflows whereas each of them exclusively quantified more than 150 proteins with significant connections to cell adhesion, extracellular matrix, and signaling pathways.

### Plasma proteome of 15 RRMS patients by LC-MS/MS

To study the RRMS proteome changes, we selected 20 samples; 5 of these were from the same HCs that were previously used for method testing and 15 were from RRMS patients at the time of diagnosis (Tables S1 and S2). The plasma proteome profiles of these 20 samples were studied according to the deepest coverage procedures tested.

### RRMS proteomic profiling using the DEPL-SP3 methodology

**Figure 3.**
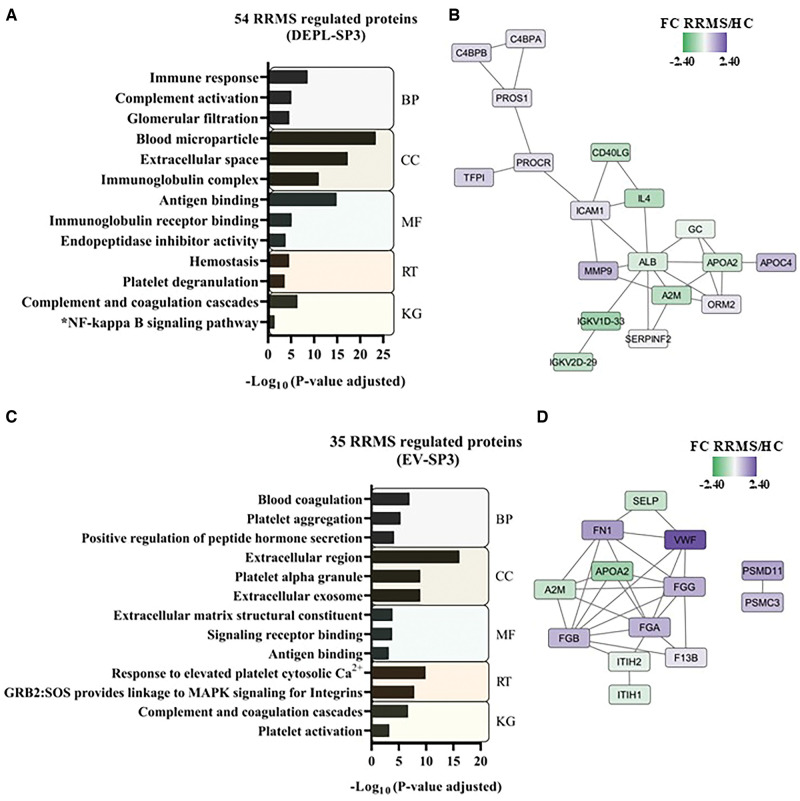
Enrichment of the GO terms, Reactome terms and KEGG pathways of RRMS regulated proteins identified in the DEPL-SP3 (A) and in the EV-SP3 (C) workflow. Analysis of protein-protein interaction (PPI) networks of the 54 and 35 RRMS regulated proteins according to the DEPL-SP3 (B) and in the EV-SP3 (D) workflow. The protein nodes are colored according to their RRMS/HC log_2_ fold change (FC). Asterisk represents significance at unadjusted *P* value < 0.05.

From an original set of 696 plasma proteins, 559 proteins were selected having 4 quantitative values in each of the groups (RRMS and HC) using the DEPL-SP3 methodology. Fifty-four proteins were found significantly regulated after performing a Welch’s *t*-test (Supplementary Data File S1). GO term enrichment analyses with these proteins showed a significant association with immune response processes (IGHV1-69, IGHV4-34, IGKV2D-29, IGKV3-20, IGKV3D-11, IGKV4-1, IGKV3D-20, JCHAIN, IGHA1, IGKV1D-33, IGKC, IGKV3D-15, IGKV3-15, IGHV3-7) and were part of hemostasis and complement/coagulation cascades (IGHG2, IGHA1, C4BPB, IGHM, C4BPA) (Figure 3A).

A STRING analysis to study PPI networks identified a cluster of 18 proteins involved in regulation of immune effector processes (Figure 3B). One of the cluster proteins, α-1-acid glycoprotein 2 (ORM2), modulates the activity of the immune system during acute-phase reactions and it was found to be upregulated in the RRMS group.^16^ Beside PPI networks, other upregulated proteins of potential involvement in RRMS were identified: catalase (CAT), CMP-N-acetylneuraminate-beta-galactosamide-alpha-2,3-syalyltransferase (ST3GAL4), and receptor-type tyrosine-protein phosphatase eta (PTPRJ) (Table 1). ST3GAL4 is involved in the synthesis of a major nerve myelin component whereas PTPRJ modulates prolongation of synaptic signaling.^17^^,^^18^ Supplementary Data File S1 lists the regulated RRMS proteins found after sample processing with the DEPL-SP3 workflow.

**Table 1.** List of regulated proteins in RRMS patients compared with healthy controls potentially associated with neuronal transmission and neural immune system.

Protein identifier	Gene name	FC RRMS/HC	Protein description
P04040	CAT	0.408	Promotes growth of T-cells
P09172	DHB	−0.642	Regulates neurotransmitters such dopamine and noradrenaline
P19652	ORM2	0.297	Modulates the activity of the immune system during acute-phase reaction
P63241	EIF5A	−0.408	Mediates effects of polyamines on neuronal process extension and survival
Q11206	ST3GAL4	1.227	Synthesizes ganglioside LM1, a major structural component of peripheral nerve myelin
Q12913	PTPRJ	0.269	Dephosphorylates PLCG1 and LAT to down-regulate prolongation of synaptic signaling
P02671	FGA	1.004 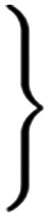	May facilitate the immune response via both innate and T-cell mediated pathways
P02675	FGB	0.958	
P02679	FGG	1.059	
P04275	VWF	2.400	Part of a biomarker signature for the diagnosis of clinically defined MS
P81605	DCD	−0.664	Promotes survival of neurons

Protein identifiers above and below the black line were detected with the DEPL-SP3 and EV-SP3 workflows, respectively.

### RRMS proteomic profiling using the EV-SP3 methodology

The isolation of plasma EV identified 1316 proteins by LC-MS/MS although a dataset of 760 proteins was selected after filtering out those proteins with less than 4 quantitative values in each group. Supplementary Data File S2 lists the regulated RRMS proteins found after sample processing with the EV-SP3 workflow. Thirty-five proteins were found significantly regulated after performing a Welch’s *t*-test. GO term enrichment analyses showed a significant association with blood coagulation processes (FGA, F13B, FGB, FGG) and with extracellular matrix structural constituent, signaling receptor binding (FGA, FN1, A2M, ANG, FGB, FGG, APOA2) and antigen binding molecular function terms (Figure 3C). The regulated proteins enriched with this workflow were primarily located in the extracellular region and in the extracellular exosome, as expected. PPI network analyses identified a cluster of 11 proteins associated with extracellular matrix structural constituent and a small cluster of 2 proteins (26S proteasome non-ATPase regulatory subunit 11, PSMD11, and 26S proteasome regulatory subunit 6A, PSMC3) involved in proteasome and prion disease (Figure 3D). Apart from the upregulated fibrinogen molecules that seem to increase innate and T-cell mediated immune responses, a high expression of the von Willebrand factor (VWF) in the 11-proteins cluster was observed. Interestingly, VWF took part of a biomarker signature for the diagnosis of clinically defined MS cases.^19^ Beside PPI networks, dermcidin (DCD), a protein which promotes survival of neurons and displays phosphatase activity was downregulated in the RRMS group^20^ (Table 1).

Both DEPL-SP3 and EV-SP3 datasets of regulated proteins were compared with a Venn diagram and only 4 proteins were found overlapped (Figure S3): α-2-macroglobulin (A2M), immunoglobulin kappa variable 3-15 (IGKV3-15), apolipoprotein A-II (APOA2), and hypoxia up-regulated protein 1 (HYOU1). The low number of overlapping features pointed to the necessity to include different workflows to increase the proteomic characterization of plasma proteins.

## DISCUSSION

The current standard procedure for MS diagnosis is based on the detection of 2 demyelinating events separated in time and in different parts of the CNS using MRI^21^ and the detection of non-specific oligoclonal IgG in CSF.^22^ However, oligoclonal bands are not exclusive to MS patients.^23^ Therefore, the emergence of molecular biomarkers capable of improving the diagnosis, monitoring and treatment of MS becomes necessary. Recently, several LC-MS/MS studies have identified increased levels of integrin subunit alpha X (ITGAX), fatty acid-binding protein 5 (FABP5), CD99 antigen (CD99), sex hormone-binding globulin (SHBG), and apolipoprotein C-III (APOC3) in CSF samples from MS patients.^24^^,^^25^

Although CSF samples have frequently been used for studies of biomarker discovery in the past, the invasive nature of the CSF extracting procedure has encouraged the employment of other biofluids which can be obtained with less interfering approaches. Moreover, a recent study of the proteome of plasma and CSF EVs from RRMS patients showed the presence of small and large CNS-derived EVs into peripheral blood suggesting their ability to cross altered blood brain barrier (BBB).^26^ Therefore, the use of plasma for the study of MS and other neurological diseases might represent an efficient alternative to the extraction and use of CSF samples.

In order to achieve higher plasma proteome coverage than that obtained with methods that use raw plasma, this study has added a depletion step of highly abundant proteins, using commercial Top 14 depletion mini spin columns, followed by the SP3 strategy achieving a greater proteome quantification (1.7× more proteins) than the SP3 approach alone. The nearly 2-fold increase of quantified proteins using the depletion devices before SP3 has previously been reported in other studies.^27^ The improvement of proteome coverage is related to the resin capacity of removing approximately 75% of albumin, although the remaining 25% of albumin and its possible protein complexes might still interfere in the detection of low abundance proteins. Furthermore, we have introduced another pre-processing step by ultracentrifugation enrichment of EVs and obtained an even better protein coverage (923 ± 130). Together, the DEPL-SP3 and EV-SP3 identified most of the plasma proteome described with the use of the different methodologies although each of them uniquely characterized different protein subsets. While the DEPL-SP3 identified cell adhesion mediators, transmembrane kinase receptors and proteins involved in axonal fasciculation, the EV-SP3 identified GTP-binding, cytoskeleton, and neutrophil degranulation proteins besides a different subset of proteins involved in cell adhesion processes (Figure 2C). Our results have demonstrated that the SP3 workflow with raw plasma allowed the best proteome coverage among the sample preparation methods that do not require an additional enrichment step. However, the use of immunodepleting columns and EV enrichment by ultracentrifugation resulted in an advantageous 1.7× and 2.4× increase of quantified proteins. Although all testing samples were from female patients, the number of protein identifications according to each methodology should not be affected by the sex of the patient.

Because of the proteome coverage depth and exclusive proteome characterizations, both methods were selected for the characterization of 15 RRMS plasma proteomes. The DEPL-SP3 and EV-SP3 workflows identified 54 and 35 differentially expressed proteins in RRMS plasma samples, respectively. Only four of them (A2M, IGKV3-15, APOA2, and HYOU1) were identified in both approaches. Four other regulated proteins—ST3GAL4, PTPRJ, VWF, and DCD—found in one of the workflows were proposed as new potential diagnostic RRMS biomarkers. Notably, VWF, which modulates the BBB permeability in MS-like lesions, was found to significantly discriminate clinically definite MS (CDMS) from non-MS neurological conditions.^19^ The increased expression of VWF observed in RRMS plasma in this study might explain the reported reduced abundance in the CSF of CDMS patients. In another investigation, VWF was increased in the plasma of mice subjected to spontaneous intracerebral hemorrhage.^28^ Moreover, mice treated with VWF showed more severe BBB damage and neuronal injury compared with control animals. Compared to other neurodegenerative disorders, ST3GAL4 gene expression played a crucial role in modulating epilepsy and anxiety/depression through its expression in thalamic neurons.^29^ Regarding inflammatory processes, VWF, DCD, and PTPRJ have been identified as atherosclerosis biomarkers.^30–32^ Therefore, distinction of RRMS-specific biomarkers from those to other neurodegenerative or inflammatory becomes difficult. RRMS biomarker specificity would be addressed in additional biomarker validation studies that might include patient samples from similar neurodegenerative and inflammatory disorders to RRMS.

Although plasma biomarker research by LC-MS/MS-based proteomics has been increasingly implemented in various disciplines of MS clinical research,^33^ a recent study has used LC-MS/MS in the analysis of tears from patients with MS. It was found out that proteins such as haptoglobin (HP), prosaposin (PSAP), cytoskeletal keratin type I (KRT1), pre-mRNA-processing factor 17 (CDC40), neutrophil gelatinase-associated lipocalin (LCN2), and phospholipase A2 (PLA2G4A) were downregulated and that cystatin C (CST3), phospholipid transfer protein (PLTP), transcobalamin-1 (TCN1), immunoglobulin lambda variable 1-47 (IGLV1-47), lactoperoxidase (LPO), and ferroptosis suppressor protein 1 (AIFM2) were upregulated in MS patients.^34^ Taking together, these studies bring the necessity to further optimize and simplify methodologies for biofluid LC-MS/MS-based proteomics to become a major diagnostic tool of neurological diseases.

## CONCLUSION

In this study, we tested 5 sample preparation methods with human plasma samples. The DEPL-SP3 and EV-SP3 methods outperformed SP3, iST, and ENRICH-iST in the quantification of proteins and peptides. EV-SP3 provided with the deepest plasma proteome coverage that did not fully overlap with the one observed with the DEPL-SP3 strategy. These results show that the proteomics analysis of complex protein matrixes such as plasma might require protocols that separately identify soluble and EV-derived biomarkers. Among ST3GAL4, PTPRJ, VWF, and DCD candidates identified with the use of both workflows with plasma samples, VWF represents a strong diagnostic RRMS biomarker that deserves validation in large external cohorts comprising patients of both sexes.

## Supplementary Material

nlaf145_Supplementary_Data

## Data Availability

The LC-MS/MS raw files and Spectronaut output files corresponding to the method testing part and to the proteomic profiling of RRMS and HC individuals of this study have been deposited to the ProteomeXchange consortium via the PRIDE partner repository with dataset identifier PXD057402 and PXD057428, respectively.
