# Supplementary material for: Identification of diagnostic biomarkers for relapsing-remitting multiple sclerosis in plasma by mass spectrometry-based proteomics
Source: J Neuropathol Exp Neurol. 2025 Dec 22;85(7):768–76. doi: 10.1093/jnen/nlaf145 (PMC13293268; doi:10.1093/jnen/nlaf145)
Supplement: nlaf145_Supplementary_Data [file nlaf145_Supplementary_Data.zip › Supplementary Figure 3.docx]

**Supplementary Figure 3**

**
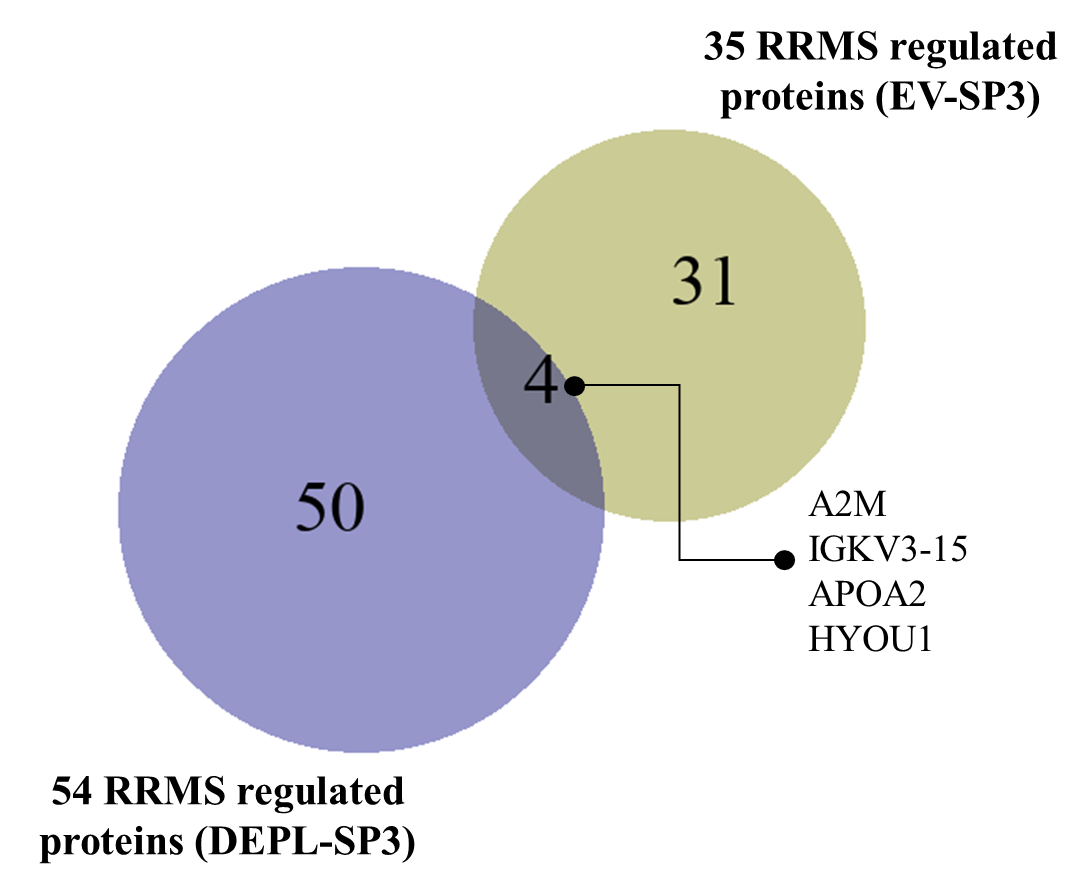
**

Comparison of the RRMS regulated proteins detected with the DEPL-SP3 and EV-SP3 methodologies. Only four proteins were identified with the two procedures.
