# Supplementary material for: Identification of diagnostic biomarkers for relapsing-remitting multiple sclerosis in plasma by mass spectrometry-based proteomics
Source: J Neuropathol Exp Neurol. 2025 Dec 22;85(7):768–76. doi: 10.1093/jnen/nlaf145 (PMC13293268; doi:10.1093/jnen/nlaf145)
Supplement: nlaf145_Supplementary_Data [file nlaf145_Supplementary_Data.zip › JNEN-25-350 Supplementary Table 1.docx]

**Supplementary Table 1.** RRMS samples used in this study with the age of the patients, the age of diagnosis and the date of extraction

| **Samples** | **Age** | **Age of diagnosis** | **Extraction Date** |
| --- | --- | --- | --- |
| RRMS 1 | 41 | 38 | 19/02/21 |
| RRMS 2 | 41 | 38 | 09/04/21 |
| RRMS 3 | 32 | 29 | 16/07/21 |
| RRMS 4 | 39 | 32 | 24/09/21 |
| RRMS 5 | 38 | 35 | 24/09/21 |
| RRMS 6 | 26 | 23 | 21/10/21 |
| RRMS 7 | 51 | 48 | 26/11/21 |
| RRMS 8 | 29 | 26 | 26/11/21 |
| RRMS 9 | 34 | 32 | 13/12/21 |
| RRMS 10 | 50 | 48 | 26/01/22 |
| RRMS 11 | 63 | 61 | 16/02/22 |
| RRMS 12 | 31 | 29 | 22/02/22 |
| RRMS 13 | 54 | 52 | 25/02/22 |
| RRMS 14 | 49 | 47 | 29/07/22 |
| RRMS 15 | 28 | 26 | 30/08/22 |
