# Supplementary material for: Identification of diagnostic biomarkers for relapsing-remitting multiple sclerosis in plasma by mass spectrometry-based proteomics
Source: J Neuropathol Exp Neurol. 2025 Dec 22;85(7):768–76. doi: 10.1093/jnen/nlaf145 (PMC13293268; doi:10.1093/jnen/nlaf145)
Supplement: nlaf145_Supplementary_Data [file nlaf145_Supplementary_Data.zip › JNEN-25-350 Supplementary Table 2.docx]

**Supplementary Table 2.** Healthy control (HC) samples used in this study with the age of the subjects and the date of extraction

| **Samples** | **Age** | **Extraction Date** |
| --- | --- | --- |
| HC 1 | 51 | 14/02/22 |
| HC 2 | 30 | 07/03/22 |
| HC 3 | 25 | 20/06/22 |
| HC 4 | 36 | 21/11/22 |
| HC 5 | 27 | 22/02/19 |
