# Supplementary material for: Identification of diagnostic biomarkers for relapsing-remitting multiple sclerosis in plasma by mass spectrometry-based proteomics
Source: J Neuropathol Exp Neurol. 2025 Dec 22;85(7):768–76. doi: 10.1093/jnen/nlaf145 (PMC13293268; doi:10.1093/jnen/nlaf145)
Supplement: nlaf145_Supplementary_Data [file nlaf145_Supplementary_Data.zip › Supplementary Figure 1.docx]

**Supplementary Figure 1**

**
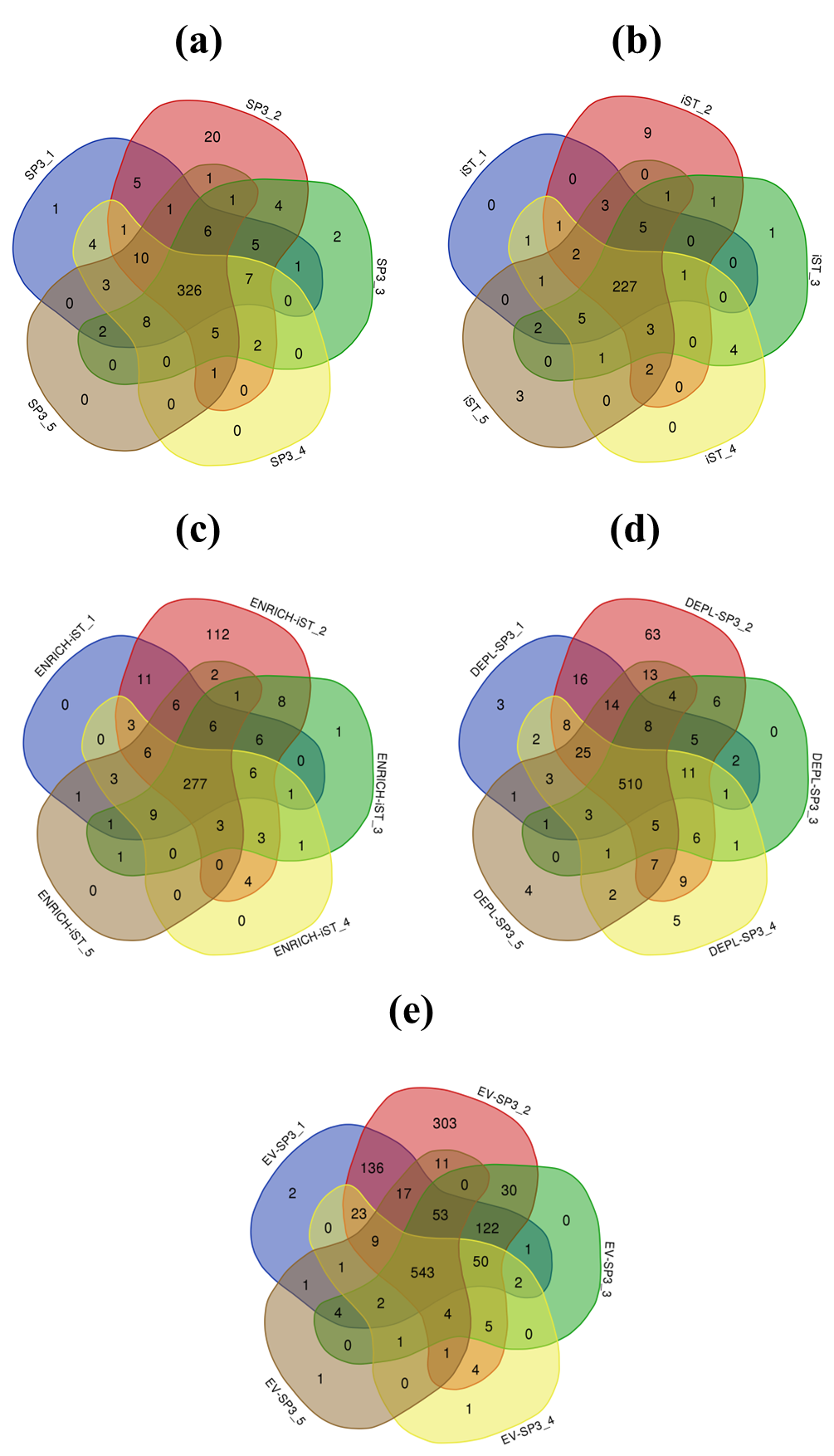
**

Venn diagrams of the proteins identified and quantified in five proteome replicates using SP3 (**a**), iST (**b**) and ENRICH-iST (**c**) with raw plasma; SP3 with highly-abundant-proteins depleted plasma, DEPL-SP3 (**d**), and SP3 with plasma extracellular vesicles, EV-SP3 (**e**).
