# Supplementary material for: Identification of diagnostic biomarkers for relapsing-remitting multiple sclerosis in plasma by mass spectrometry-based proteomics
Source: J Neuropathol Exp Neurol. 2025 Dec 22;85(7):768–76. doi: 10.1093/jnen/nlaf145 (PMC13293268; doi:10.1093/jnen/nlaf145)
Supplement: nlaf145_Supplementary_Data [file nlaf145_Supplementary_Data.zip › Supplementary Figure 2.docx]

**Supplementary Figure 2**


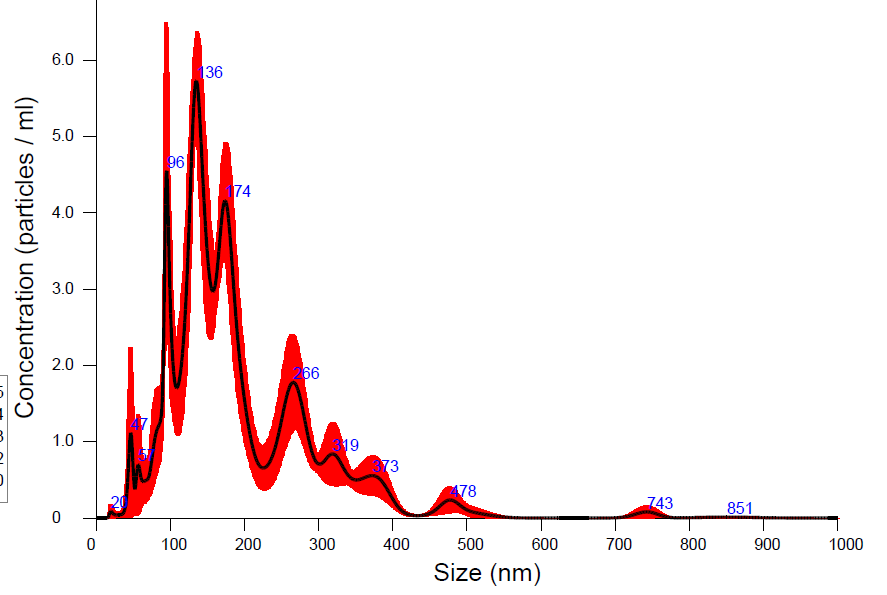


Nanoparticle tracking analysis (NTA) shows a relative enrichment of larger particles (mean size of 192 nm) in the ultracentrifugated EV fraction.
